# Efficient Active Oxygen Free Radical Generated in Tumor Cell by Loading-(HCONH_2_)·H_2_O_2_ Delivery Nanosystem with Soft-X-ray Radiotherapy

**DOI:** 10.3390/ma11040596

**Published:** 2018-04-12

**Authors:** Lei Xu, Yiran Shao, Chengkang Chang, Yingchun Zhu

**Affiliations:** 1 School of Materials Science and Engineering, Shanghai Institute of Technology, Shanghai 201499, China; 156081122@mail.sit.edu.cn; 2 Key Laboratory of Inorganic Coating Materials CAS, Shanghai Institute of Ceramics, Chinese Academy of Sciences, Shanghai 200050, China; shaoyiran@mail.sic.ac.cn; 3 Shanghai Innovation Institute for Materials, Shanghai University, Shanghai 200444, China; 4 University of Chinese Academy of Sciences, Beijing 100049, China

**Keywords:** formamide peroxide, ROS, soft-X-ray radiotherapy, tumor hypoxia

## Abstract

Tumor hypoxia is known to result in radiotherapy resistance and traditional radiotherapy using super-hard X-ray irradiation can cause considerable damage to normal tissue. Therefore, formamide peroxide (FPO) with high reactive oxygen content was employed to enhance the oxygen concentration in tumor cells and increase the radio-sensitivity of low-energy soft-X-ray. To improve stability of FPO, FPO is encapsulated into polyacrylic acid (PAA)-coated hollow mesoporous silica nanoparticles (FPO@HMSNs-PAA). On account of the pH-responsiveness of PAA, FPO@HMSNs-PAA will release more FPO in simulated acidic tumor microenvironment (pH 6.50) and subcellular endosomes (pH 5.0) than in simulated normal tissue media (pH 7.40). When exposed to soft-X-ray irradiation, the released FPO decomposes into oxygen and the generated oxygen further formed many reactive oxygen species (ROS), leading to significant tumor cell death. The ROS-mediated cytotoxicity of FPO@HMSNs-PAA was confirmed by ROS-induced green fluorescence in tumor cells. The presented FPO delivery system with soft-X-ray irradiation paves a way for developing the next opportunities of radiotherapy toward efficient tumor prognosis.

## 1. Introduction

Radiotherapy (RT) is still an important and efficient treatment for most patients with clinical tumor [1]. However, due to the characteristics of tumor recurrence, it is necessary to completely kill tumor cells by repeated RT with super-hard X-ray, which has serious adverse effects on normal tissues [2]. RT can make interstitial fluid generate free radicals (such as O^2−^, H_3_O^+^, HO·, H·, etc.) in the human body, and these free radicals react with biological macromolecules, resulting in irreversible DNA damage [3]. Nowadays, it is highly warranted to strengthen the efficient killing of irradiation on tumor tissues and reduce the damage to normal tissues. The soft X-ray radiation that we developed produces negligible damage to normal tissue, and the devices that excite soft X-ray radiation are less expensive than the conventional RT devices.

**Scheme 1:**
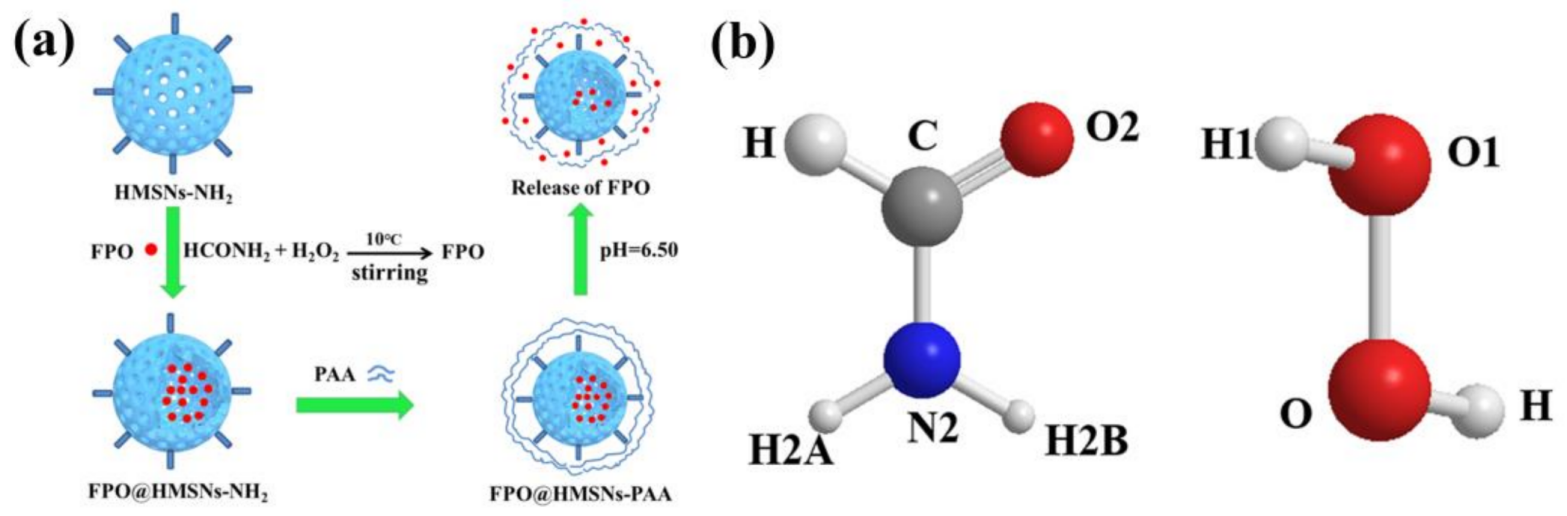
(**a**) Schematic illustration of the preparation of FPO@HMSNs-PAA and the stimuli-responsive release of formamide peroxide (FPO) from FPO@HMSNs-PAA in response to pH 5.0; (**b**) the structure of FPO.

Oxygen, playing an important role in RT, can combine with the free radicals to generate new and toxic organic peroxide free radicals (ROO·) [4,5]. However, one important factor to limit radiotherapeutic efficacy is that intracellular microenvironment of most solid tumors is significantly hypoxic [6,7,8]. In order to overcome tumor hypoxia, many researches depend on transporting radiosensitizers, such as O_2_, reactive oxygen species (ROS), and radiosensitive drugs (such as bleomycin [9], topotecan [10], artemisinin [11], etc.) to the tumor hypoxic regions by the drug delivery system (DDS) [12]. Hydrogen peroxide adducts, such as sodium percarbonate and sodium perborate, are very important fine chemical products in the chemical industry and they have broad application prospects in agriculture, industry, and medicine [13,14]. However, some inorganic peroxides have the characteristics of slight toxicity and low reactive oxygen content, thus limiting their application. Thus, a non-toxic organic peroxide (formamide peroxide) with high active oxygen content was found, which rapidly dissolved in water and produced a large amount of oxygen. When compared with other inorganic peroxides, formamide peroxide (FPO) has higher active oxygen content owing to the presence of two forms of hydrogen bonding between formamide and hydrogen peroxide: (1) O–H…O, O1–H1…O2; (2) N–H…O, N2–H2A…O1, N2–H2B…O1 (showed in Scheme 1b). An infinite one-dimensional structure of FPO is formed by O1–H1…O2 and N2–H2A…O1, while a two-dimensional structure formed by N2–H2B…O1. Then, a three-dimensional super-molecule structure of FPO that is finally obtained is similar to percarbamide’s [15]. FPO rapidly dissolves in water to produce hydrogen peroxide and decomposes further into oxygen. Therefore, our study is devoted to employing FPO to enhance the oxygen content in tumor cells and to further increase the radio-sensitivity of soft-X-ray; however, it has not been reported by any research. 

With the rapid development of nanotechnology, it presents a great potential to enhance radiotherapeutic efficacy and minimize the side effects by using targeted drug delivery system (DDS) [16]. A wide variety of inorganic nanoparticles, such as layered double hydroxide nanoparticles (LDHs) [17], mesoporous silica nanoparticles (MSNs) [18,19], mesoporous TiO_2_ nanoparticles (MTNs) [20], and others, are employed as drug nanocarriers to improve the bioavailability of therapeutic drugs and avoid the decomposition of drugs before they reach the tumor tissue. Among them, MSNs with characteristic features of stable mesopores, large surface areas, adjustable pore size, good biocompatibility, and abundant silanol groups (Si–OH) on the surface of nanoparticles have been used to fabricate stimuli-responsive DDS (such as redox- [21], pH- [22], enzyme- [23], and light-responsive release systems [24]). Many previous studies have showed that stimuli-responsive MSNs can efficiently transport therapeutic drugs into tumor cells for improving radio-/chemotherapeutic effects. According to a recent reference, copper-impregnated MSNs (Cu-MSNs) loading a catalase inhibitor was used to enhance the levels of ROS in tumor cells and further killing cancer cells [25]. Therefore, MSNs can be the best choice for one-demand DDS. When compared with MSNs, huge hollow cavities with high drug-loading capacities make hollow mesoporous silica nanoparticles (HMSNs) an ideal carrier for the soluble peroxide [26,27,28].

In this work, FPO as ROS resource was utilized to increase the oxygen concentration in tumor cell, which was synthesized in the cavity and channels of HMSNs (FPO@HMSNs) through a chemical reaction process between formamide and hydrogen peroxide at room temperature. To enhance the stability of FPO and control the release of FPO, FPO@HMSNs was capped with a pH-responsive PAA layers (FPO@HMSNs-PAA). Enhanced FPO release occurred in simulated acidic tumor media (pH 6.50) and subcellular endosomes (pH 5.0), whereas reduced release occurred in simulated normal tissues (pH 7.40). When compared with the radiation that was used in previous studies, the use of soft X-rays with lower intensity has negligible radiation effects on cells. When exposed soft-X-ray irradiation, released FPO generates abundant ROS to mediate tumor cells death, and the presence of ROS in tumor cells was verified by observing the stronger green fluorescence that was produced by the reaction between ROS and DCFH-DA. The strategy combining FPO and soft-X-ray irradiation will provide new insight into the development of radiotherapy sensitization.

## 2. Materials and Methods

Mouse breast cancer cell lines 4T1 were obtained from the cell bank of the Chinese Academy of Sciences (Shanghai, China).

### 2.1. Preparation of HMSNs

According to previous reports, mono-dispersed hollow mesoporous silica nanoparticles were synthesized by a typical synthesis procedure [29,30,31]. Firstly, the solid silica dioxide nanoparticles (sSiO_2_) were synthesized using the StÖber method as a hard template for the next sculpture. In brief, a mixture, including NH_3_·H_2_O (3.14 mL), ethanol (71.4 mL), and deionized water (10 mL) was stirred at 30 °C, and TEOS (6 mL) was rapidly added. After stirring for 2 h, the precipitate (sSiO_2_) was collected by centrifugation, washed with ethanol and distilled water, and dried in vacuum, respectively. Secondly, the core/shell nanoparticles (m SiO_2_@SiO_2_) were synthesized. As-prepared sSiO_2_ (0.5 g) was dispersed into deionized water (100 mL) under ultrasound for 20 min. Then, CTAB (0.75 g), ethanol (150 mL), deionized water (150 mL), and NH_3_·H_2_O (2.75 mL) were added into the above sSiO_2_ suspension. After stirring for 2 h at room temperature, TEOS (1.5 mL) was rapidly added and the reaction was maintained for a further 6 h. The white product (m SiO_2_@SiO_2_) was centrifuged and washed several times with deionized water. Third, HMSNs were synthesized by a selective etching approach. mSiO_2_@ SiO_2_ was dispersed into a Na_2_CO_3_ solution (50 mL, 0.4 M) and the mixture continuously reacted at 50 °C for 10 h. After being centrifuged and washed with ethanol and deionized water, the precipitate was lyophilized. Finally, an ion-exchange method was used to remove the templates CTAB in the mesoporous channels. The crude product was suspended into a mixture of NH_3_·H_2_O/C_2_H_5_OH (0.3 g, 180 mL) and was stirred at 50 °C for 6 h, which should be repeated three times to entirely remove CTAB. Then, the white product, denoted as HMSNs, was collected by centrifugation.

### 2.2. Fabrication of FPO@HMSNs-PAA/FPO@HMSNs-PAA-FITC

FPO-loaded and PAA-coated HMSNs (FPO@HMSNs-PAA) were prepared as follows: Firstly, amino-functionalized HMSNs were synthesized according to a previous report [32]. In detail, the HMSNs (0.5 g) were dispersed into toluene (50 mL) containing APTES (0.4 mL). After refluxing at 80 °C for 8 h, the precipitate was centrifuged and rinsed with ethanol for several times. The product denoted as HMSNs-NH_2_ was dried in a vacuum. Next, FPO was synthesized in the cavity and the channels of HMSNs-NH_2_. As-prepared HMSNs-NH_2_ (0.4 g) was dispersed into deionized water (15 mL) and then formamide (4 g) was added. After stirring for 24 h at the room temperature, H_2_O_2_ (30% W, 13 g) was added dropwise to the above mixture solution and the reaction lasted for another 48 h at 10 °C. Finally, FPO@HMSNs-PAA was synthesized by adding PAA (0.5 g) into the above mixture. After being stirred 40 min, the product was collected by centrifugation and freeze-dried. As the control experiment, the encapsulation of FPO in naked HMSNs (FPO@HMSNs) and the coating of PAA on naked HMSNs (HMSNs-PAA) were prepared with a similar process.

At the same time, FPO@HMSNs-PAA-FITC was achieved by the following process: The FITC-APTES was prepared by the reaction between FITC (15 mg) and APTES (40 μL) in methanol (6 mL) for 24 h under dark conditions. Then, the obtained FPO@HMSNs-PAA (50 mg) was dispersed into the FITC-APTES solution and was stirred for another 24 h in the same condition. After collected by centrifugation, FPO@HMSNs-PAA-FITC was washed with methanol and dried in vacuum.

### 2.3. Evaluation of FPO Loading Capacity and pH-Responsive Release Profiles in Different Buffer Solutions

In order to indirectly calculate the amount of FPO that was incorporated in FPO@HMSNs-PAA and FPO@HMSNs, the quantity of H_2_O_2_ needs to be calculated by the principle that the reaction between H_2_O_2_ and KMnO_4_ in sulphuric acid medium quantitatively [33]. Firstly, we used the UV/Vis spectrophotometer to measure a series of different H_2_O_2_ concentrations by the change value in the KMnO_4_ solution (λ = 525 nm). Then, FPO@HMSNs-PAA (5 mg) was added into the KMnO_4_ solution, thus allowing them to stand in the dark. After 30 min, we obtained the absorbance value of KMnO_4_ solution. By the relationship between H_2_O_2_ and KMnO_4_, we calculated the amount of H_2_O_2_ and indirectly calculated the amount of FPO in FPO@HMSNs-PAA. The amount of FPO in FPO@HMSNs was obtained by a similar procedure. We calculated the FPO loading capacity as the following equation:

FPO loading capacity (%, *w*/*w*) = mass of FPO/mass of FPO@HMSNs-PAA

The evaluation of FPO releasing profiles was similarity to calculate the FPO loading capacity, which was performed as follows: the as-prepared FPO@HMSNs and FPO@HMSNs-PAA were both dissolved in different PBS (pH 7.40, 6.50, and 5.0) with accurate weight. Then, we took the supernatant (10 mL) to add in as-prepared the mixture solution including KMnO_4_ and H_2_SO_4_. This process would continue for about 2 h, and the KMnO_4_ absorbance value of mixture solution was detected by the UV/Vis spectrophotometer (λ = 525 nm) every 5 min. Finally, the release curves were plotted using several data points that were obtained above.

### 2.4. Measurement of ROS Produced by FPO@HMSNs-PAA under Soft-X-ray Irradiation

ROS produced by FPO@HMSNs-PAA under soft-X-ray irradiation was measured by the fluorescence spectrometer (HORIBA Jobin Yvon, Paris, France). Firstly, FPO@HMSNs-PAA with the FPO concentration of 0, 8 and 16 μg·mL^−1^ were dispersed in 10 mL PBS (pH 6.50, 7.40) including pure terephthalic acid (PTA, 8.31 mg) and NaOH (4 mg) [34]. The mixture solution was exposed to diverse soft-X-ray irradiations with the currents of 6 mA, 8 mA, and 10 mA and a voltage of 55 kV. After 2 min, the fluorescence spectra of solution were obtained by the fluorescence spectrometer (λ = 310 nm). At the same time, the fluorescence spectra of control groups without soft-X-ray irradiation were measured.

### 2.5. Intracellular Uptake of FPO@HMSNs-PAA

To observe general distribution of nanoparticles within tumor cells, the confocal laser scanning microscopy (CLSM) was used to trace the endocytosed nanoparticles according to previous reports [35]. Briefly, 4T1 cells were seeded in petri dishes and incubated overnight at 37 °C. After being incubated with FPO@HMSNs-PAA-FITC (20 μg·mL^−1^) for 12 h, the medium was discarded and the cells were washed with PBS to remove the residual nanoparticles. Then, **4**′,**6**′-diamidino-**2**-phenylindole (DAPI, 0.5 mL) in methanol (10%) was added and was incubated for 15 min to stain the nuclei and fix the cells. Subsequently, the staining solution was discarded and Typan Blue (2 mg·mL^−1^ PBS) was introduced into the petri dishes to quench intracellular fluorescence for 2 min. Then the cells were washed with PBS and methanol, and Buffer A solution was added into the petri dishes. Finally, CLSM was employed to obtain the images of the intracellular uptake of FPO@HMSNs-PAA.

### 2.6. In Vitro Cytotoxicity Assay of FPO@HMSNs-PAA with and without Soft-X-ray Irradiation

The cytotoxicity of the nanoparticles, including free HMSNs, FPO, HMSNs-PAA, and FPO loaded NPs were quantitatively investigated by using MTT reduction assay. Firstly, 4T1 cells were seeded into 96-well plates at a density of 5.0 × 10^4^ cells per well and were cultured in the DMEM medium supplemented with 10% FBS and 1% penicillin streptomycin combination at 37 °C in a humid 5% CO_2_ atmosphere. After being incubated 24 h, the culture media was replaced with fresh media consisting of free FPO or FPO@HMSNs-PAA with equivalent FPO concentration (8, 16, 32, and 40 μg·mL^−1^). When co-incubated for another 24 h, the mixed medium was discarded and washed with PBS (pH 7.40). Afterwards, MTT/PBS (20 μL, 5 mg·mL^−1^) was prepared and added in the each well to quantify the living cells. After another co-incubation for 4 h, the mixture was discarded and dimethyl sulfoxide (DMSO, 150 μL per well) was added. Gently shaken 5 min, the 96-well plates were measured on a microplate reader (Thermo Scientific, Waltham, MA, USA) at a wavelength of 492 nm. When considering the comparison, the cytotoxicity of FPO-free HMSNs and HMSNs with a concentration equal to FPO@HMSNs-PAA (20, 40, 80, and 100 μg·mL^−1^) was evaluated with a similar protocol.

To research the radio-sensitizing effect of FPO@HMSNs-PAA with soft-X-ray irradiation, the prepared cells were incubated with FPO@HMSNs-PAA at different FPO concentrations (0, 8, 16, 32, and 40 μg·mL^−1^) for another 2 h. Then, the 96-well plates were exposed to soft-X-ray irradiation with the current of 6, 8, and 10 mA and the voltage of 55 kV for 2 min. Subsequently, the cells were cultured for 24 h and the MTT assay was used to monitored the absorbance of each well. As the control group, the cytotoxicity of 4T1 cells without soft-X-ray was determined by the same procedure. Finally, the cell cytotoxicity was expressed as the percentage of cell viability in contrast to the control cells.

### 2.7. Measurement of ROS in Tumor Cell

Oxidatively sensitive non-fluorescence probe DCFH-DA was utilized to measure the intracellular ROS level. DCFH-DA can be deacetylated by nonspecific esterase to form DCFH, which was further oxidized by intracellular ROS to produce a stable fluorescent ROS-sensitive compound **2**′,**7**′-dichlorofluorescein (DCF) [36]. In this study, 4T1 cells were seeded into petri dishes at a density of 5.0 × 10^4^ cells and cultured with serum-free media containing FPO@HMSNs-PAA (16 and 32 μg·mL^−1^). After a co-incubation for 2 h, the cells were exposed on soft-X-ray irradiation (55 kV, 8 mA) for 2 min. Then, another 4 h co-incubation, the cell medium was discarded and washed with serum-free medium. Subsequently, DCFH-DA (10 μM) was added and incubated for 30 min at 37 °C. The cells were washed with PBS and fluorescence microscopy was used to observe the green fluorescence. Meanwhile, the above cell was harvested and accurately counted for additional measurement. Then, the aliquots of cells were re-suspended in fresh medium without serum. Finally, fluorescence spectrometer intracellular (HORIBA Jobin Yvon, Paris, France) was employed to measure the fluorescence spectra of DCF, which reflected the ROS level f in 4T1 cells. At the meantime, the control groups without soft-X-ray irradiation were accomplished.

## 3. Results and Discussion

### 3.1. Preparation and Characterization of FPO@HMSNs-PAA

The preparation of FPO@HMSNs-PAA included four steps. Firstly, HMSNs were prepared by using a selective etching strategy. Secondly, APTES was conjugated onto HMSNs (HMSNs-NH_2_) for reacting with PAA. Then, FPO was synthesized in the cavity and channels of HMSNs-NH_2_ by the reaction of formamide and hydrogen peroxide (FPO@HMSNs-NH_2_) at 10 °C. Finally, PAA was coated onto FPO@HMSNs-NH_2_ (FPO@HMSNs-PAA) for a pH-responsive release of guests. The whole procedure is illustrated in Scheme 1a.

**Figure 1:**
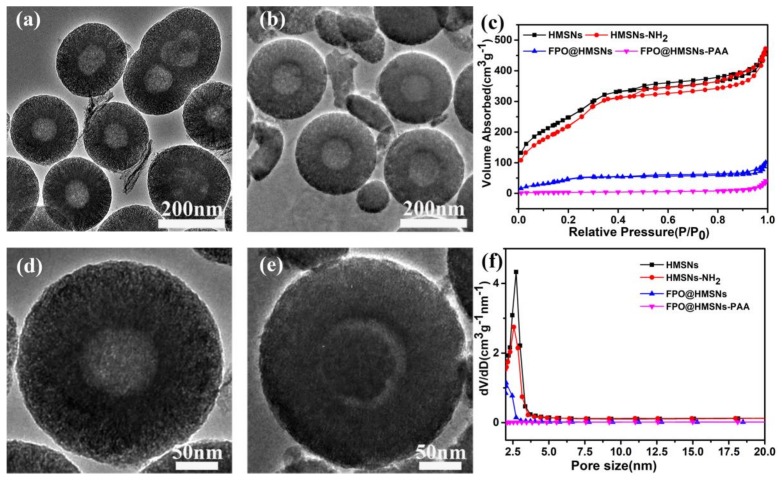
TEM images of hollow mesoporous silica nanoparticles (HMSNs) (**a**, **d**); HMSNs-polyacrylic acid (PAA) (**b**) and FPO@HMSNs-PAA (**e**). Adsorption-desorption isotherms of HMSNs, HMSNs-NH_2_, FPO@HMSNs and FPO@HMSNs-PAA (**c**). Pore size distributions nitrogen of HMSNs, HMSNs-NH_2_, FPO@HMSNs and FPO@HMSNs-PAA (**f**).

**Figure 2:**
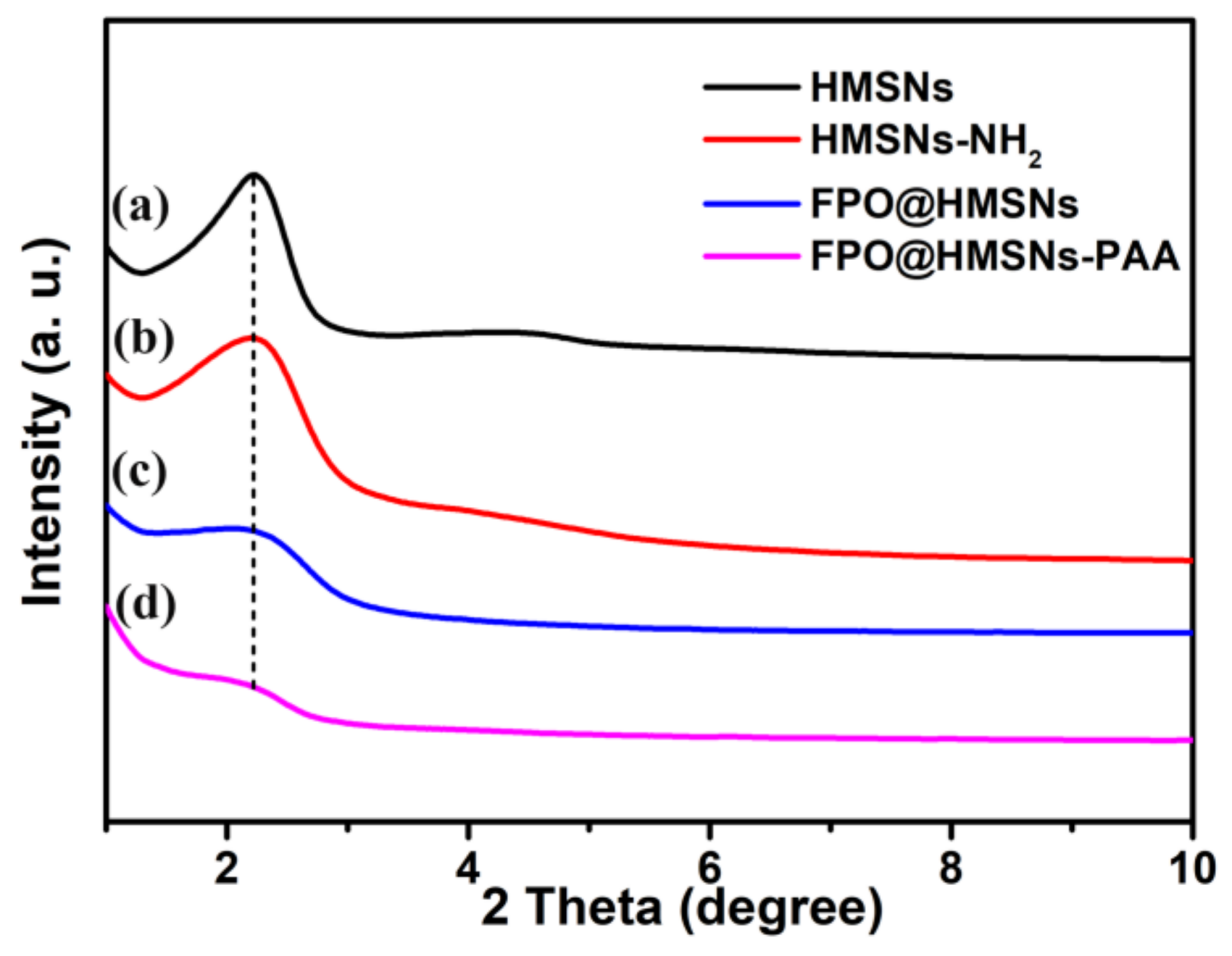
Low-angle XRD patterns of (**a**) HMSNs; (**b**) HMSNs-NH_2_; (**c**) FPO@HMSNs; and (**d**) FPO@HMSNs-PAA.

The morphology and size of samples were obtained by TEM. The prepared HMSNs exhibited a uniform hollow structure and well-order mesoporous shell with an average diameter of 300 nm (Figure 1a). The amplified TEM image in Figure 1d shows that the average thickness of the shell is about 80 nm. When compared with smooth surface of HMSNs, the surface of HMSNs-PAA (Figure 1b,e) was rough, indicating that the PAA as “gatekeepers” was successfully coated on the surface of HMSNs. The Figure 1e shows that FPO was successfully synthesized in the cavity of HMSNs. As shown in the low-angle XRD patterns (Figure 2a,b), the diffraction peak at 2θ = ca. 2.271 points that HMSNs and HMSNs-NH_2_ have an ordered mesoporous structure, while the intensity of the diffraction peaks decreased, owing to the successful synthesis of FPO in HMSNs (Figure 2c,d). 

The N_2_ adsorption-desorption isotherms and pore size distribution curves of HMSNs, HMSNs-NH_2_, FPO@HMSNs, and FPO@HMSNs-PAA were measured by N_2_ adsorption-desorption analysis. As shown in Figure 1c, the as-prepared HMSNS presents a typical Langmuir type IV isotherm with sharp capillary condensation, which demonstrates a well-defined mesoporous structure. When compared with HMSNs, the FPO@HMSNs-PAA had a lowered P/P_0_ and hardly presented relative pressure leap. 

The BET surface area (S_BET_), pore volume (V_pore_), and pore size distribution (D_pore_) are summarized in Table 1. The SBET and V_pore_ of HMSNs were measured as 1166 m^2^·g^−1^ and 0.84 cm^3^·g^−1^, which was reduced to 282 m^2^·g^−1^ and 0.28 cm^3^·g^−1^ after FPO loading, with a further reduction to 94 m^2^ ·g^−1^ and 0.13 cm^3^·g^−1^ after PAA coating. The D_pore_ of HMSNs was about 2.7 nm and remained constant at 2.5 nm in HMSNs-NH_2_, while the D_pore_ of FPO@HMSNs-PAA was hardly detected after the FPO loading and PAA coating (Figure 1f). All of the above results manifest that FPO@HMSNs-PAA has been successfully fabricated.

**Figure 3:**
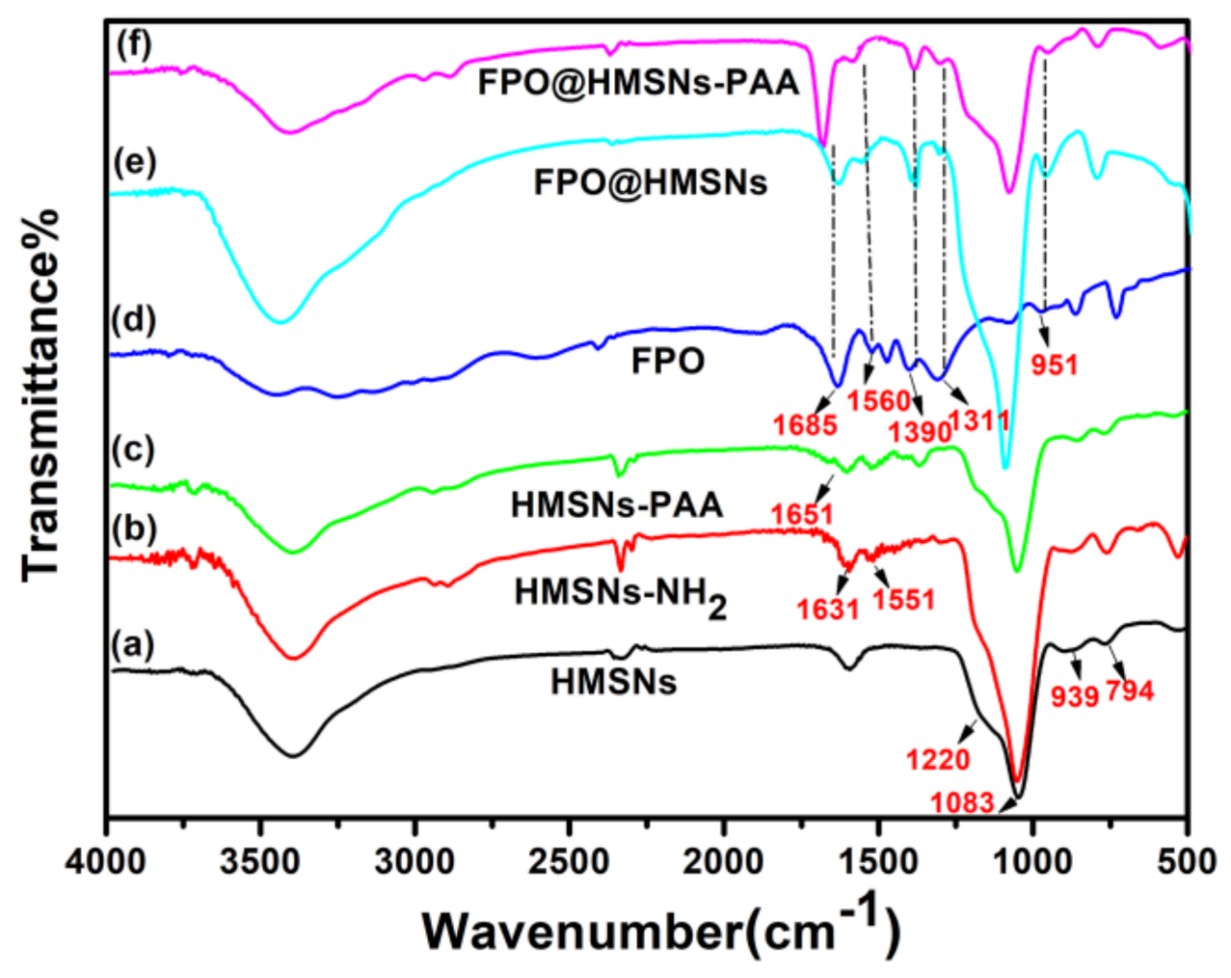
Fourier transform infrared (FTIR) spectra of (**a**) HMSNs; (**b**) HMSNs-NH_2_; (**c**) HMSNs-PAA; (**d**) FPO; (**e**) FPO@HMSNs and (**f**) FPO@HMSNs-PAA.

The successful fabrication of FPO@HMSNs-PAA was verified by other different methods. As shown in Table 1, the zeta potential of HMSNs sample is −33.7 mV. After HMSNs reacted with APTES, the HMSNs-NH_2_ exhibited a positive potential of about 36.1 mV due to the amino-groups (–NH_2_) on the surface of HMSNs. The zeta potential of FPO@HMSNs-PAA further decreased to −56.5 mV, indicating that PAA has been successfully coated on the surface of HMSNs. The fourier transform infrared (FTIR)with low energy range (2000–400 cm^−1^) was used to examine chemical structures of HMSNs, HMSNs-NH_2_, HMSNs-PAA, FPO and FPO@HMSNs-PAA. As shown in Figure 3a, as-prepared HMSNs exhibit the characteristic bands of silica at 1220, 1083, 939, and 794 cm^−1^, which is due to the asymmetric stretching vibrations of Si–O–Si at 1220 and 1083 cm^−1^. The stretching vibration of Si–OH was at 939 cm^−1^, while the symmetric stretching vibration of Si–O–Si was at 794 cm^−1^ [37]. When compared with HMSNs, the HMSNs-NH_2_ showed new peaks at 1631 and 1551 cm^−1^ (Figure 3b), which are attributed to the stretching vibration of amide I and amide II [38,39]. In Figure 3c, the appearance of the peak at 1651 cm^−1^ is due to the carbonyl group (ν_C=O_ = 1600~1850 cm^−1^), indicating the formation of HMSNs-PAA [33]. Figure 3d shows the characteristic absorption peaks of FPO, which exhibits a whole train of peaks at 2981, 1685, 1560, 1390, 1311, and 951 cm^−1^. Judging by the position of these peaks that appeared, the observed bonds were assigned to the carbon hydrogen bond (δ_C–H_ at 1390 cm^−1^), the carbonyl group (ν_C=O_ at 1685 cm^−1^) and the amide (δ_N–H_ at 1311 cm^−1^) [40]. The peak at 1560 and 951 cm^−1^ is attributed to the absorption of H_2_O_2_ [41]. The IR spectrum of FPO@HMSNs (Figure 3e) and FPO@HMSNs-PAA (Figure 3f) were almost consistent with that of native FPO (Figure 3d), manifesting the presence of FPO in these nano-carriers. Furthermore, the increased peak intensity of carbonyl group also indicates that FPO is successfully synthesized in the cavity of HMSNs and FPO@HMSNs–PAA is successfully fabricated.

### 3.2. Evaluation of FPO Loading Capacity and pH-Responsive Release Profiles in Different Buffer Solution (pH 5.0, 6.50 and 7.40)

**Figure 4:**
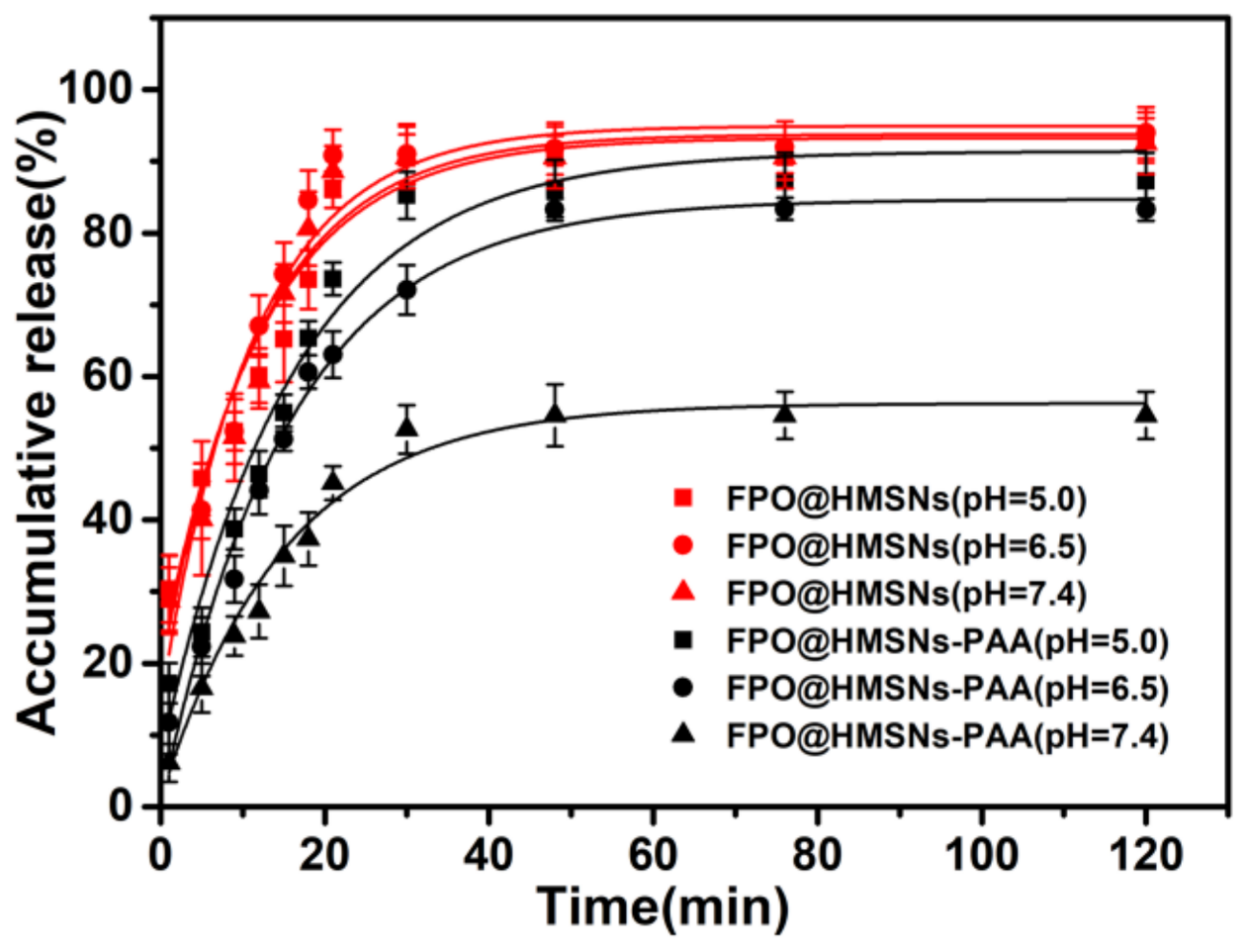
FPO release profiles from FPO@HMSNs and FPO@HMSNs-PAA in different PBS solution. Compared with FPO@HMSNs, FPO@HMSNs-PAA showed pH-responsive release profiles.

To investigate the FPO loading capacity and the release properties of FPO@HMSNs and FPO@HMSNs-PAA, a UV/Vis spectrophotometer at 525 nm was utilized to detect absorbance of KMnO_4_. As a result, the FPO loading content in FPO@HMSNs was 54.90 wt %, while the loading content of FPO in FPO@HMSNs-PAA was 38.32 wt % (shown in Table 1). The cumulative FPO release from FPO@HMSNs and FPO@HMSNs-PAA nano-systems was implemented in different PBS (pH 5.0, 6.50, and 7.40), correspondingly simulating the microenvironment of subcellular endosomes, tumor cells, and the human normal tissues or blood, respectively [42,43]. Figure 4 obviously shows that FPO that was released from FPO@HMSNs had little imparity in different PBS. After 120 min, the cumulative FPO release amount was up to 92.9% at pH 5.0, 93.4% at pH 6.50, and 92.5% at pH 7.40. Whereas, FPO that was released from FPO@HMSNs-PAA was distinctly different and the release rate also changed at different pH release media. The cumulative FPO release amount was up to 87.2% at pH 5.0, 83.3% at pH 6.50, and 54.5% at pH 7.40 after 120 min. FPO@HMSNs-PAA has the regularly pH-relevant release owing to the coated layer PAA. Under the acid condition (pH 5.0, 6.50), there is the weakened electrostatic force between–NH^3+^ and–COOH. At the same time, the solubility of PAA decreases as the pH value decreases, further reducing the force between PAA and HMSNs-NH_2_ [44]. Thus, the resistance of the FPO release from nanocarriers FPO@HMSNs-PAA will diminish and FPO release rate will increase. The resistance in pH 7.40 is stronger than that in pH 6.50, which results in the FPO release rate relatively reducing.

### 3.3. ROS Generation of FPO@HMSNs-PAA under Soft-X-ray Irradiation

Water radiolysis produces some radicals, such as e_aq_^−^, O_2_·^−^, HO·, and HO_2_·^−^ [45,46,47]. Afterwards, a series of reactive oxygen radicals will generate when FPO release from FPO@HMSNs-PAA under soft X-ray irradiation. These procedures occur as follows:

H_2_O → e_aq_^−^ + H∙ + HO∙, (1)

e_aq_^−^ + H_2_O_2_ → HO∙ + OH^−^, (2)

e_aq_^−^ + O_2_ → O_2_∙^−^, (3)

H∙ + O_2_ → HO_2_∙, (4)

**Figure 5:**
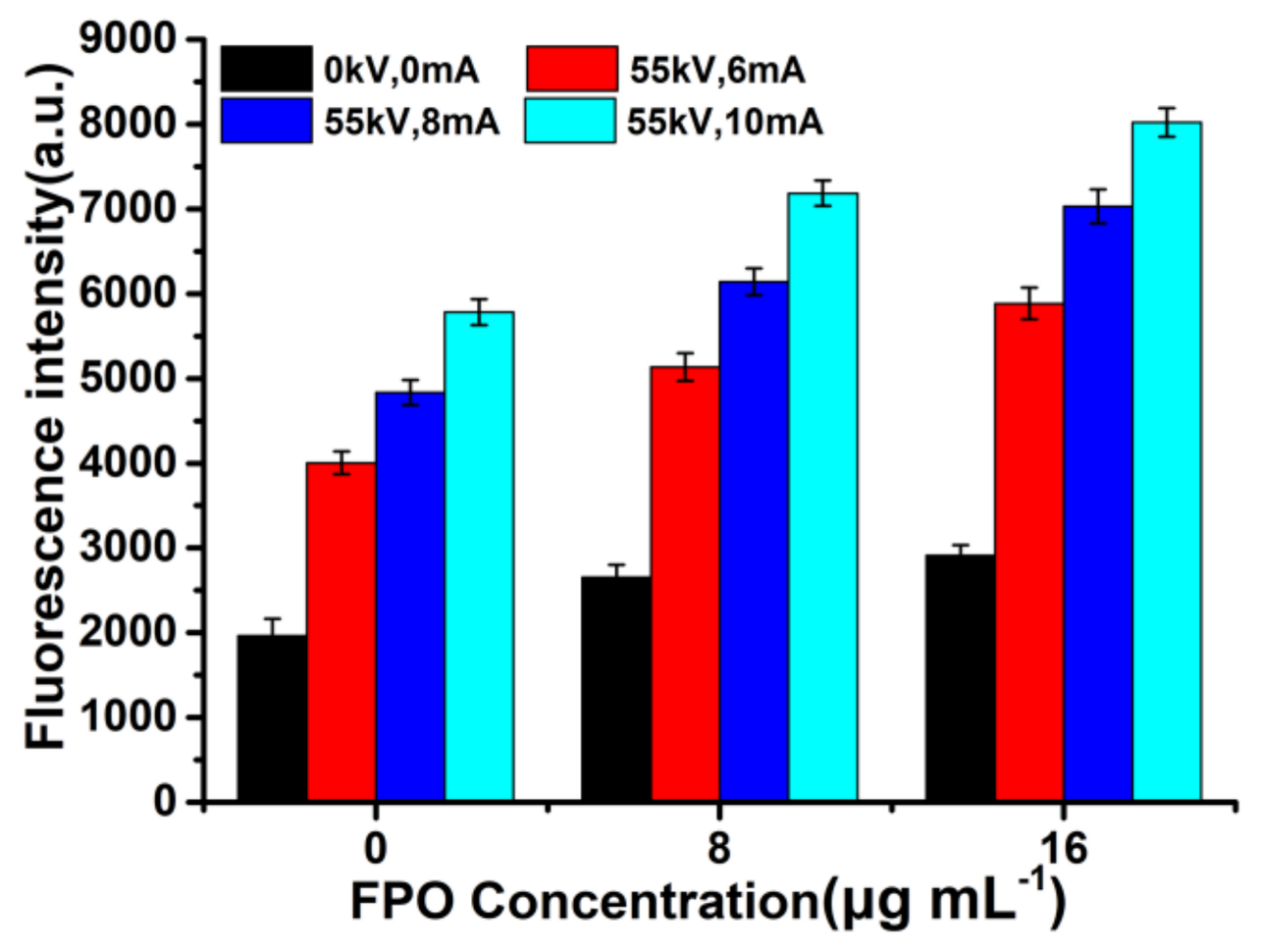
Comparison of fluorescence intensity of FPO@HMSNs-PAA (the FPO concentration of 0, 8, and 16 μg·mL^−1^) treated with different radiotherapy (RT) intensity (55 kV, 6 mA; 55 kV, 8 mA; 55 kV, 10 mA) in PBS (pH 6.50). It reflected the ROS level.

The fluorescence spectrum was employed to detect the ROS generation of FPO@HMSNs-PAA under soft-X-ray. Non-fluorescent PTA can trap with HO· to generate highly fluorescent hydroxyl (terephthalic acid) [48]. With the concentration of FPO (0, 8 and 16 μg·mL^−1^) increasing, the fluorescence intensity of the mixed solution (pH 6.50) slightly increased without soft-X-ray irradiation. FPO@HMSNs-PAA could release FPO at pH 6.50 and further decompose to form a little HO·, which makes the fluorescence intensity of the solution slightly increased. When the samples were exposed to soft-X-ray irradiation (RT), the fluorescence intensity showed in Figure 5 increased distinctly with the concentration of FPO@HMSNs-PAA increasing. When compared with the group that was treated with soft-X-ray irradiation, the experimental group treated with the combination of FPO@HMSNs-PAA and soft-X-ray showed stronger fluorescence intensity, which could be put down to the higher ROS generation. Released FPO from FPO@HMSNs-PAA could decompose H_2_O_2_ and further produce O_2_. When exposed soft -X-ray irradiation, H_2_O_2_ and O_2_ react with e_aq_^−^ to HO∙, O_2_∙^−^, and so on. More HO∙ results in higher fluorescence intensity. At the same time, the fluorescence intensity is consistent with the intensity of soft-X-ray, which increases with the intensity of RT changing from 6 mA, 55 kV to 10 mA, 55 kV.

**Figure 6:**
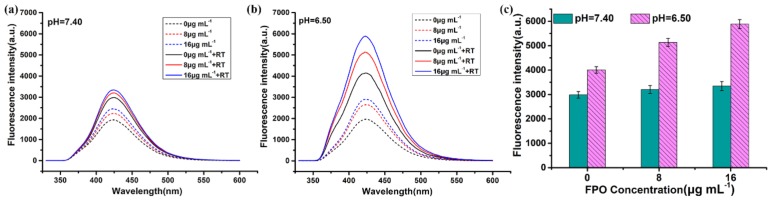
Fluorescence assay of FPO@HMSNs-PAA (the FPO concentration of 0, 8, and 16 μg mL^−1^) with same RT intensity (55 kV, 6 mA) in different PBS solution of (**a**) pH 7.40 and (**b**) pH 6.50. FPO@HMSNs-PAA at pH 6.50 showed stronger fluorescence intensity than that at pH 7.40. (**c**) Comparison of fluorescence intensity about pH 7.40 and pH 6.50 (55 kV, 6 mA).

At the same time, the fluorescence intensity of FPO@HMSNs-PAA in different PBS-PTA mixed solution (pH 7.40, 6.50) with the same concentration of FPO and the same intensity of RT (55 kV, 6 mA) were detected by the fluorescence spectrum. Figure 6 shows that FPO@HMSNs-PAA dispersed into the PBS-PTA solution with pH 7.40 (Figure 6a) displayed lower fluorescence intensity than that with pH 6.50 (Figure 6b). Figure 6c directly demonstrates that the nanocomposite can release more FPO in PBS (pH 6.50), so it generates higher ROS level when exposed to soft-X-ray irradiation. 

### 3.4. Cellular Uptake and Cytotoxicity of FPO@HMSNs-PAA 

**Figure 7:**
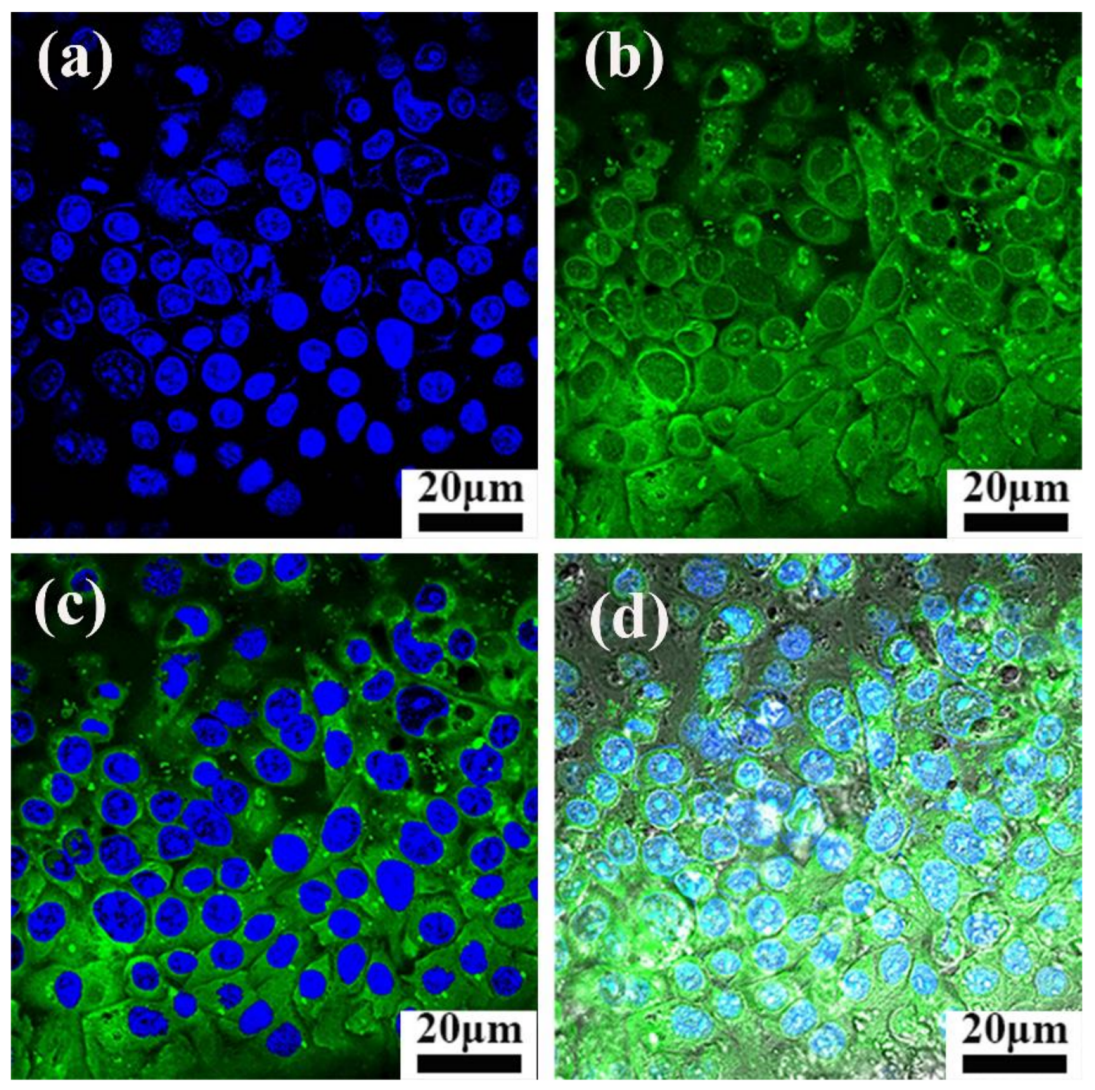
Confocal laser scanning microscopic (CLSM) image of 4T1 cells co-incubated with FPO@HMSNs-PAA-FITC. (**a**) Blue fluorescence from cell nuclei stained with **4**′,**6**′-diamidino-**2**-phenylindole (DAPI); (**b**) Green fluorescence from FPO@HMSNs-PAA-FITC; (**c**) Merged image; and, (**d**) Bright field image of overlay.

To observe the cellular uptake of nanocomposites, FPO@HMSN-PAA was conjugated to FITC, and FITC can emit green fluorescence upon excitation with light at 488 nm. The confocal laser scanning microscopy (CLSM, Olympus, Japan) images (Figure 7) show that an obvious strong green fluorescence appeared surrounding the blue nucleus, which were stained with DAPI. It indicates that FPO@HMSN-PAA can be effectively ingested by 4T1 cells. 

**Figure 8:**
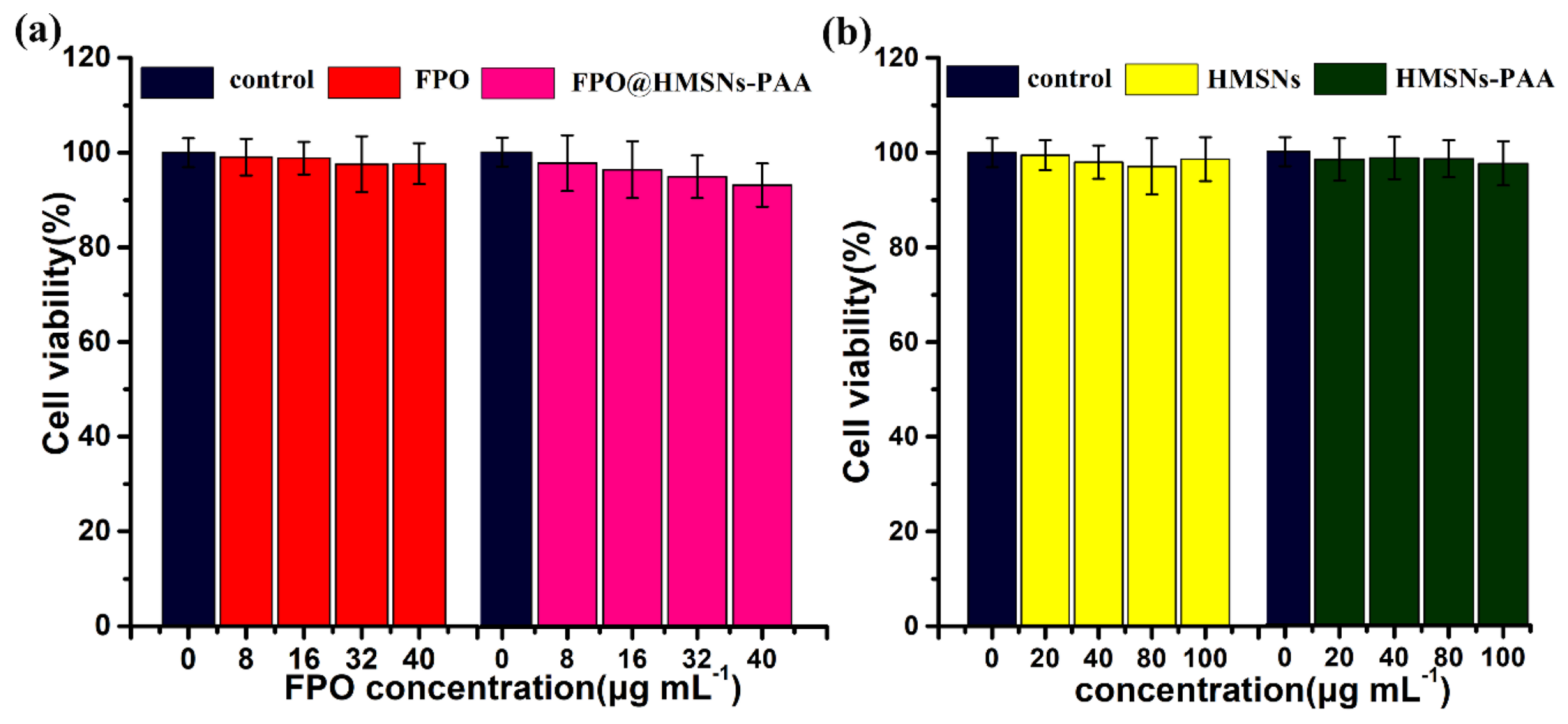
The cytotoxicity of (**a**) FPO, FPO@HMSNs-PAA; (**b**) HMSNs and HMSNs-PAA against 4T1 cells after 24 h co-incubation. When compared with the control group, all of the samples showed negligible cytotoxicity. Data were represented as means ± SDs (*n* = 6).

To evaluate the pharmacological activity of free FPO and FPO released from the nanocarriers, the cytotoxicity of 4T1 cells was investigated by MTT assay. As can be seen from the Figure 8a, the cell viability of both free FPO and FPO-loaded NPs hardly decreased with the concentration of the samples increasing. After co-culturing in DMEM media for 24 h, the cell viability was 97.5% with free FPO, and it was 94.8% with FPO@HMSNs-PAA at 32 μg·mL^−1^ of FPO. The comparable results were acquired at FPO concentrations of 8, 16, and 40 μg·mL^−1^ (Figure 8a). Because of the distinct cell uptake mechanism, the cytotoxicity was obviously different between free FPO and FPO@HMSNs-PAA. Free FPO enters cells through the passive diffusion-dependent process, which makes the effective component H_2_O_2_ decomposed rapidly before it gets to cellular microenvironment. Nevertheless, the cell uptake of FPO@HMSNs-PAA occurs by a fluid phase pinocytosis or nonspecific adsorptive endocytosis [49]. When internalized into the cells, FPO@HMSNs-PAA will release FPO and will further generate H_2_O_2_ and O_2_. Then, it forms a small amount of ROS, which will cause damage to DNA and then lead to a slightly higher cell death rate. It is known that formamide as the by-products of FPO has a little toxicity. However, formamide can be metabolized to formic acid and ammonia under acidic conditions (Figure S1f in the supplementary materials). To prove that the slight cell apoptosis was caused by ROS that was generated by FPO@HMSNs-PAA, the 4T1 cell was incubated with formamide at the concentration of 0, 8, 16, 32, and 40 μg·mL^−1^ for 24 h. In Figure S1a, the cell viability was 102.4%, 98.5%, 100.8%, and 99.7%, indicating that low concentration of formamide is not cytotoxic. It can be seen from the Figure S1b–e, the 4T1 cells well maintained morphology.

**Figure 9:**
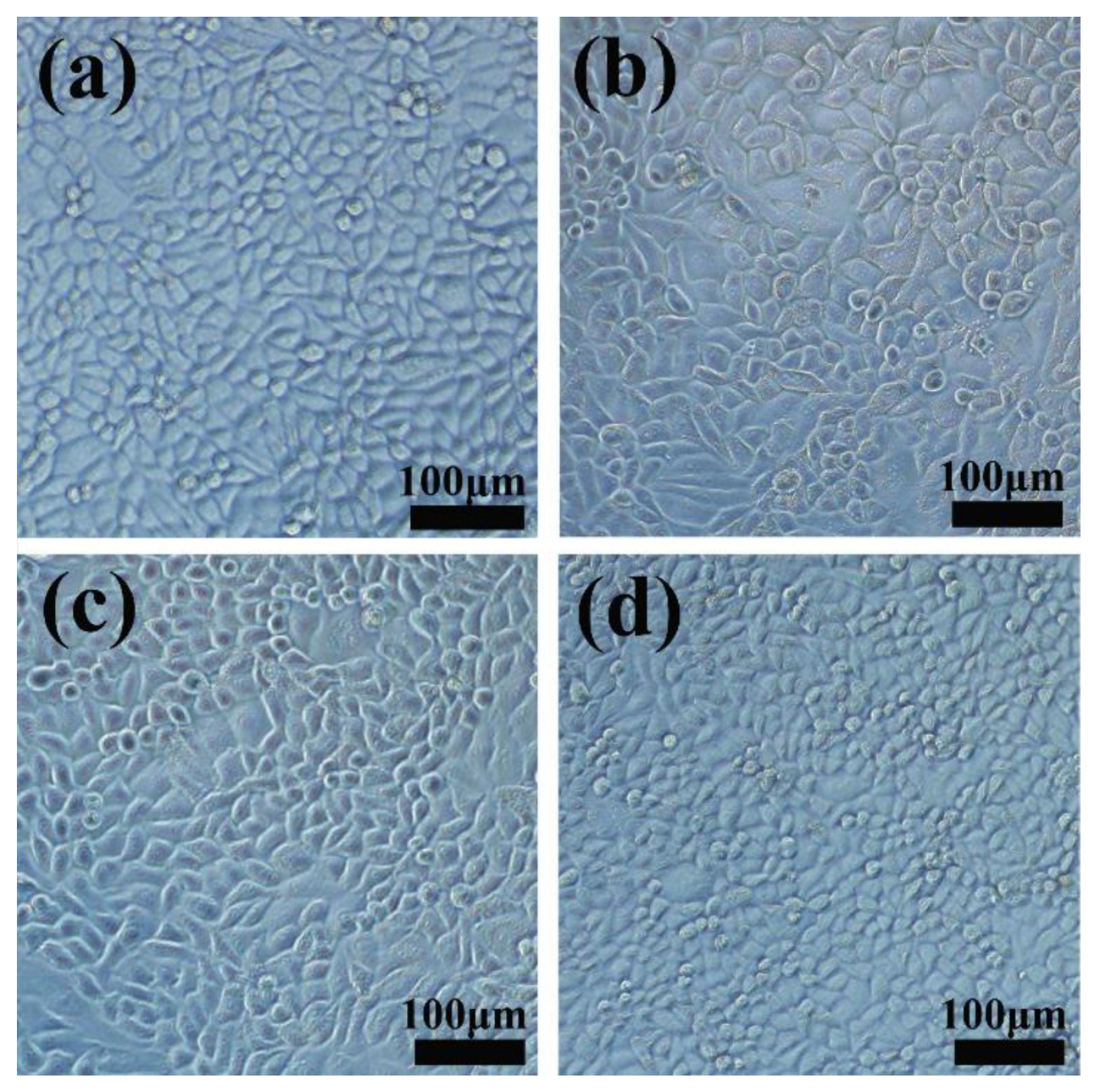
4T1 cells Microscopy images of control group (**a**); HMSNs (**b**); HMSNs-PAA (**c**) and FPO@HMSNs-PAA (**d**). The concentration HMSNs, HMSNs-PAA was 80 μg·mL^−1^, and FPO concentration was 32 μg·mL^−1^.

For comparison, the same method was used to evaluate the cytotoxicity of empty HMSNs and HMSNs-PAA with different concentration. As is shown in Figure 8b, both HMSNs and HMSNs-PAA cause little cytotoxicity against 4T1 cells, even at a concentration of up to 100 μg·mL^−1^ after co-incubation for 24 h. Here, the concentration of both HMSNs and HMSNs-PAA was equal to the FPO concentration. Furthermore, Figure 9a–d showed that cells well maintained morphology after co-culture 24 h, indicating that low-concentration HMSNs, HMSNs-PAA, and FPO@HMSNs-PAA are biocompatible with inappreciable cytotoxicity.

### 3.5. Observation of Intracellular ROS Generation by Fluorescence Microscopy

**Figure 10:**
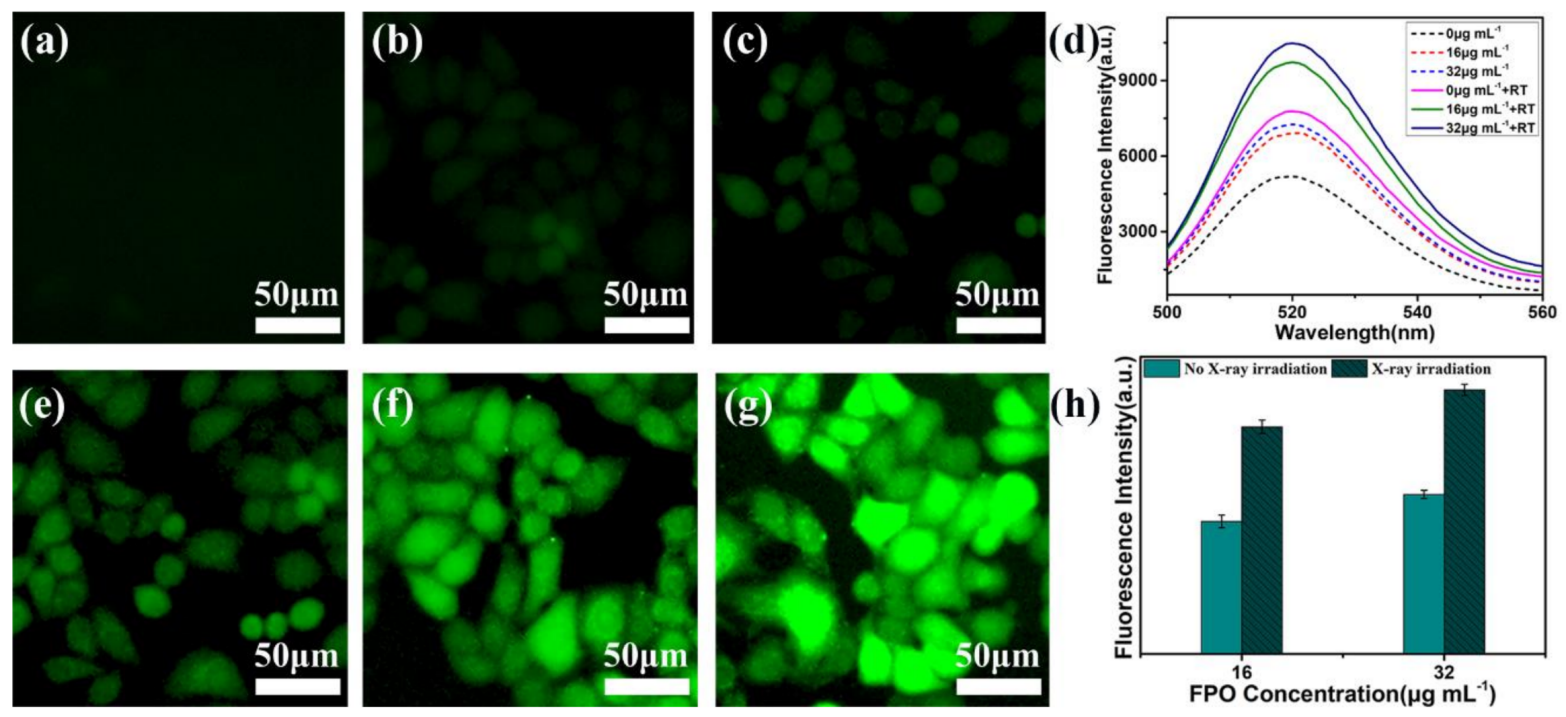
The fluorescence images of 4T1 cells with different treatment: (**a**) cell cultured with only DCFH-DA as the control group; (**b**) FPO@HMSNs-PAA (16 μg·mL^−1^) without RT; (**c**) FPO@HMSNs-PAA (32 μg·mL^−1^) without RT; (**e**) RT (55 kV, 8 mA) without sample; (**f**) FPO@HMSNs-PAA (16 μg·mL^−1^) + RT (55 kV, 8 mA); (**g**) FPO@HMSNs-PAA (32 μg·mL^−1^) + RT (55 kV, 8 mA); and, (**d**, **h**) the comparison of fluorescence intensity in 4T1 cells with different treatment.

DCFH-DA was used to evaluate the ROS level in 4T1 cells in that non-fluorescent DCFH-DA could be oxidized by intracellular ROS to form the highly fluorescent DCF [50]. DCFH-DA (10 μM) was added to the culture media without FBS after 4T1 cells were incubated with FPO@HMSNs-PAA (0, 16 and 32 μg·mL^−1^ of FPO) and were treated with or without soft-X-ray irradiation (55 kV, 8 mA). The results would be obtained by fluorescence microscopy and analyzed by fluorescence spectrophotometer. As shown in Figure 10a, the control group without FPO@HMSNs-PAA and low-intensity RT shows unnoticeable green fluorescence in cells. When treated with low-intensity RT, the group without FPO@HMSNs-PAA exhibits slightly green fluorescence due to ROS that is generated by water radiolysis (Figure 10e). When 4T1 cells were only treated with FPO@HMSNs-PAA (16 and 32 μg·mL^−1^ of FPO), a little green fluorescence can be observed, owing to ROS that was generated by the released FPO in cells (Figure 10b,c). As can be seen from the Figure 10f,g, the use of FPO@HMSNs-PAA, combined with soft-X-ray irradiation, leads to highest green fluorescence, indicating that this nanocomposite obviously raises the intracellular ROS level. In addition, with the concentration of FPO increased, the ROS level enhanced. Meanwhile, the fluorescence intensity of DCF in Figure 10d,h also reflects that the combination of FPO@HMSNs-PAA and RT leads to the strongest green fluorescence in cells. Thus, it is concluded that the combination of FPO@HMSNs-PAA and soft-X-ray irradiation generates a rich amount of ROS.

### 3.6. The Radio-Sensitization of FPO@HMSNs-PAA with Soft-X-ray Irradiation on Tumor Cell

**Figure 11:**
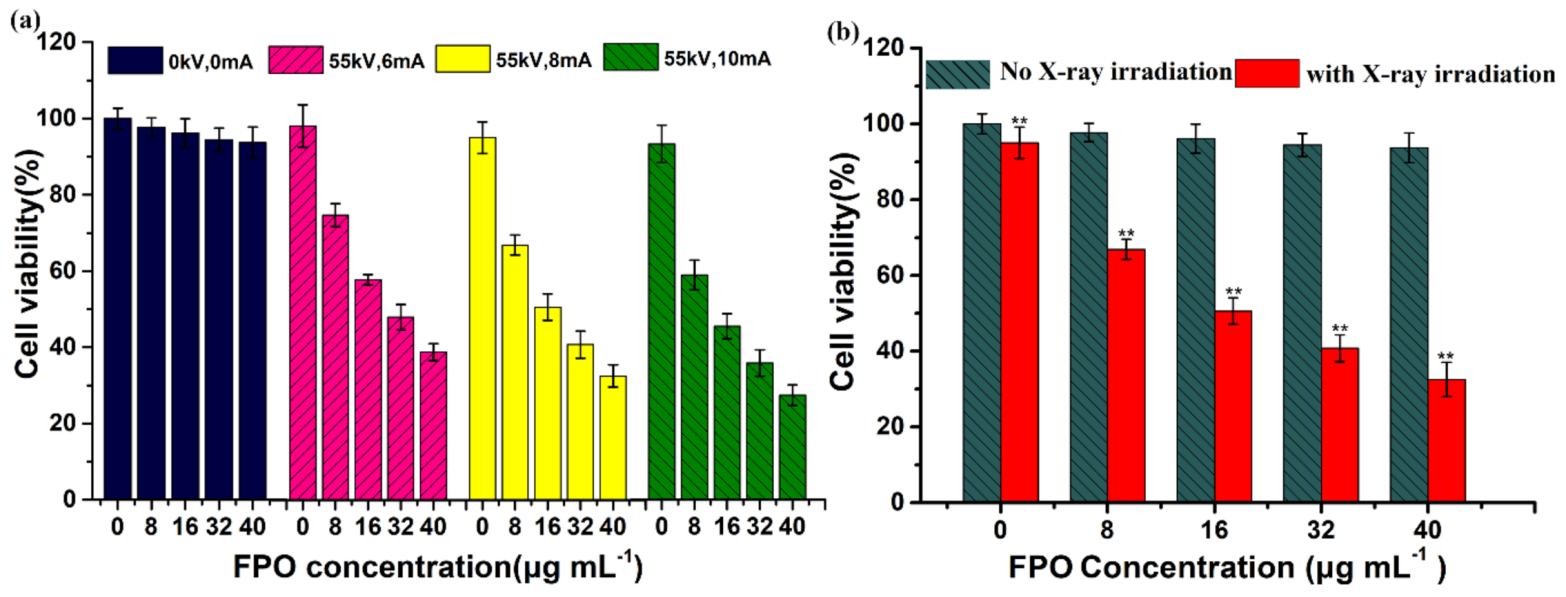
(**a**) Viability of 4T1 cell co-incubated with FPO@HMSNs-PAA (0–40 μg·mL^−1^ of FPO) after different RT treatment; (**b**) Cell viability comparison of FPO@HMSNs-PAA with (** *p* < 0.01) or without (*p* > 0.05) RT treatment (55 kV, 8 mA). Data were represented as means ± SDs (*n* = 6).

It is well known that a higher ROS level usually causes a higher cell apoptosis rate. Some studies have used copper as a catalyst to induce anti-cancer drugs, such as DOX and ascorbic acid, to generate ROS, which causes tumor cells apoptosis [17,51]. The above results have shown that low-concentration FPO@HMSNs-PAA without soft-X-ray irradiation showed no obvious cytotoxicity against 4T1 cells. To demonstrate that the combination of FPO@HMSNs-PAA and soft-X-ray enhanced cell death rate, 4T1 cells were co-incubated with FPO@HMSNs-PAA at different FPO concentrations and were given different intensity of soft-X-ray irradiation. The cell viability was detected by MTT assay in the same way. In Figure 11a, when exposed to the same voltage (55 kV) and the current of 6, 8, and 10 mA, the cell viability of the experimental group without FPO@HMSNs-PAA was 98.1%, 95.1%, and 93.3%. This result reveals that soft-X-ray used alone has no obvious cytotoxicity. However, when being co-incubated with FPO@HMSNs-PAA at a different FPO concentration, the experimental group with soft-X-ray irradiation exhibited higher cytotoxicity against tumor cells. After 24 h, the cell viability was 74.7% (6 mA), 66.9% (8 mA), and 58.9% (10 mA) at 8 μg·mL^−1^ of FPO. When the concentration of FPO was up to 32 μg·mL^−1^, the cell viability of 4T1 cells decreased to 47.9%, 40.8%, and 35.9%, respectively. The cell viability reduced with the intensity of soft-X-ray irradiation increasing. All of the results indicate that nanocomposites FPO@HMSNs-PAA with soft-X-ray irradiation surmount the shortcoming of high-energy X-ray irradiation and achieve the effect of radiotherapy sensitization.

**Figure 12:**
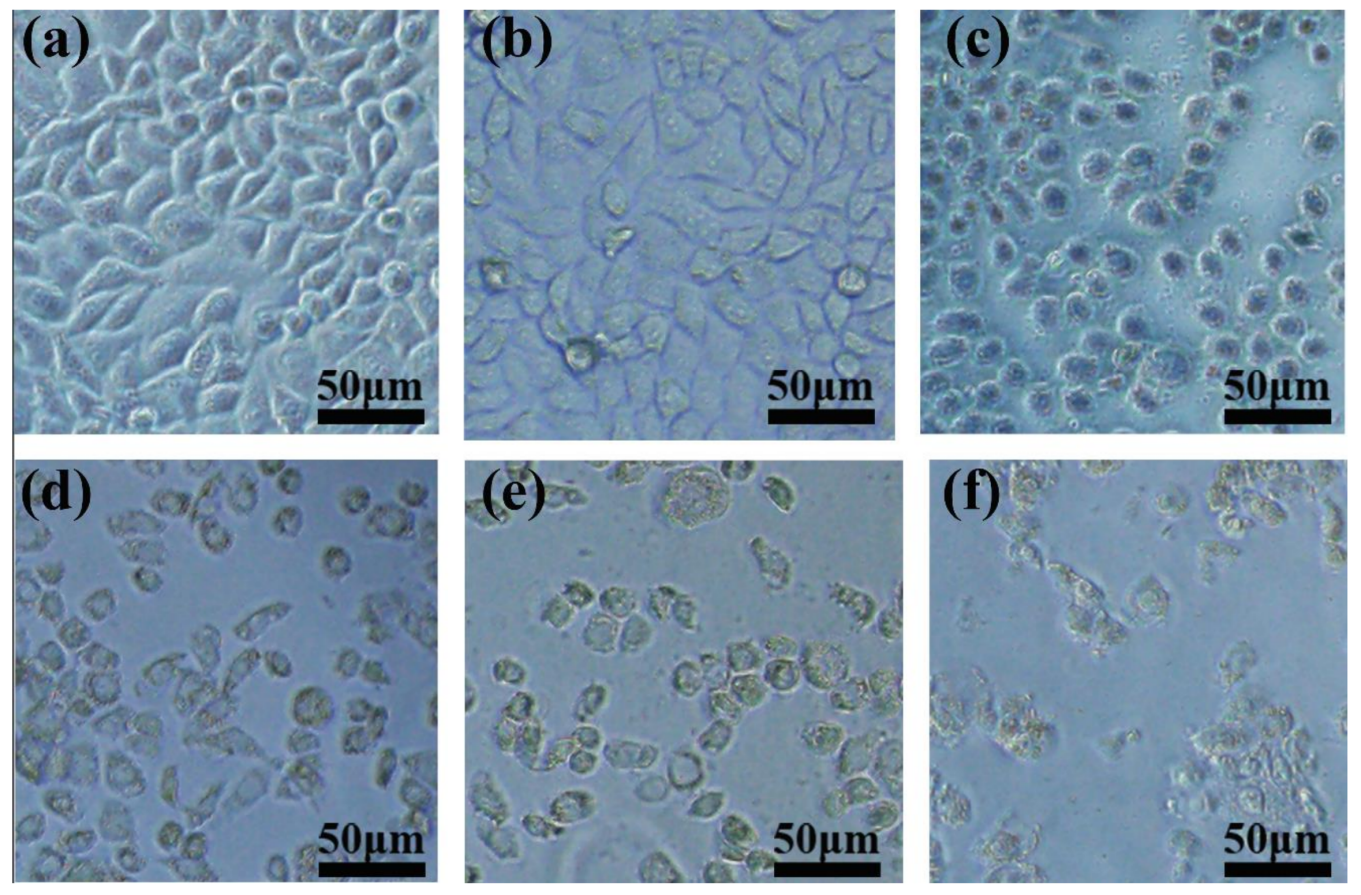
(**a**) Control group without sample and RT; (**b**) FPO@HMSNs-PAA (0 μg·mL^−1^) + RT (55 kV, 8 mA); (**c**) FPO@HMSNs-PAA (8 μg·mL^−1^) + RT (55 kV, 8 mA); (**d**) FPO@HMSNs-PAA (16 μg·mL^−1^) + RT (55 kV, 8 mA); and, (**e**) FPO@HMSNs-PAA (32 μg·mL^−1^) + RT (55 kV, 8 mA); (**f**) FPO@HMSNs-PAA (40 μg·mL^−1^) + RT (55 kV, 8 mA).

By loading FPO into HMSNs-PAA, we found that the combination of FPO@HMSNs-PAA and soft-X-ray resulted in higher cell necrosis rate (Figure 11b). It has been widely accepted that ROS can damage DNA and lead to cellular necrosis. Considerable research efforts have been devoted that free radicals being generated during ionization could combine with oxygen to form toxic combinations [52]. FPO@HMSNs-PAA releases FPO in tumor cells, which generates H_2_O_2_ and further decomposes to produce O_2_. Water radiolysis generates a series of ROS, such as H· and H· can combine with O_2_ to produce HO_2_·. These free radicals cause damage to DNA and reduce the cell viability [53]. The corresponding microscopy images of 4T1 cells co-cultured with FPO@HMSNs-PAA after RT treatment have been shown in Figure 12a–f. When exposed to soft-X-ray irradiation, the morphology and amount of 4T1 cells has obviously changed and reduced. Thus, FPO@HMSNs-PAA with soft-X-ray irradiation can provide a more advanced way for killing the tumor cells and improving the radio-therapeutic effects.

## 4. Conclusions

**Scheme 2:**
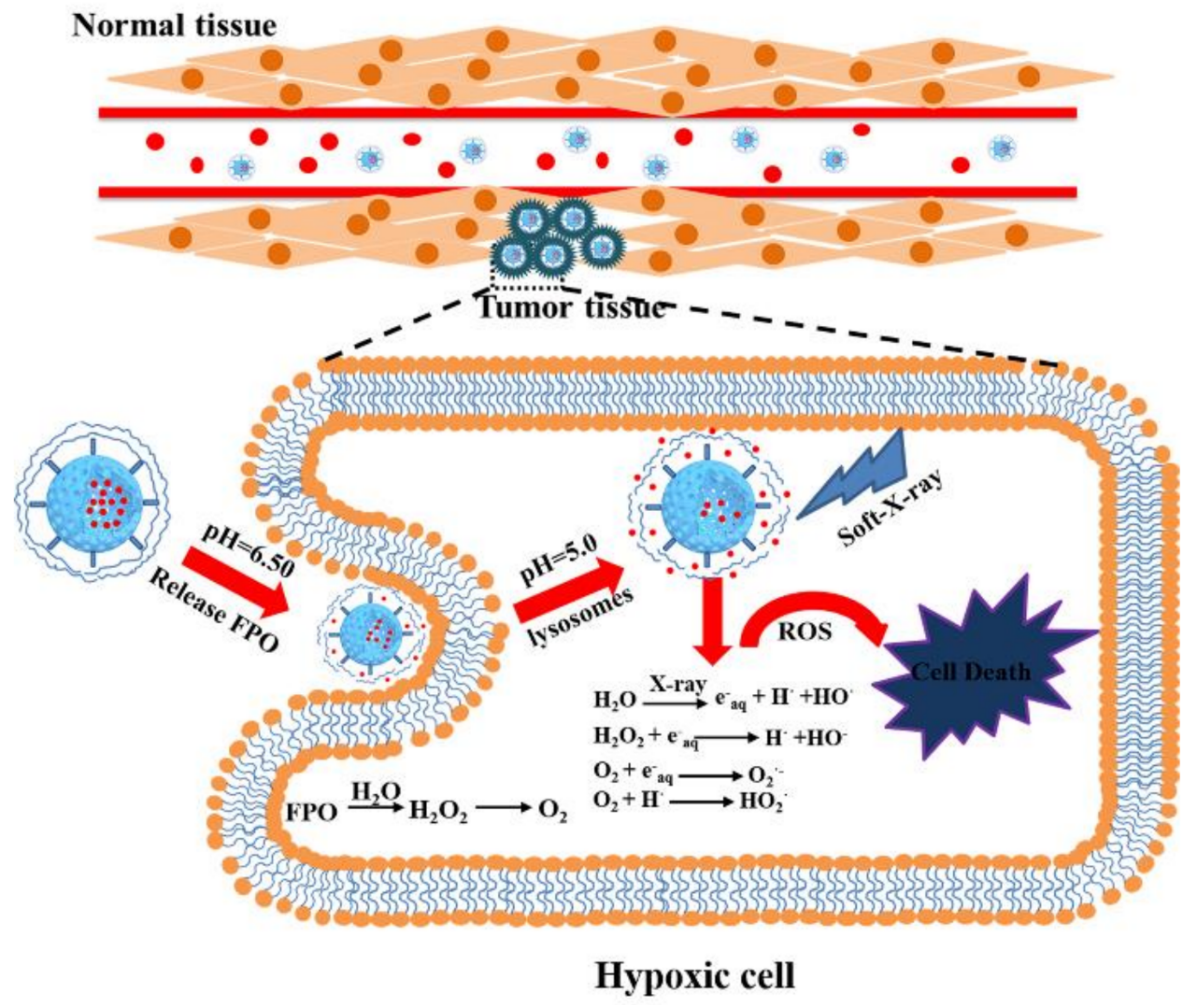
Schematic illustration of the procedure of tumor cells death after treated with FPO@HMSNs-PAA and soft-X-ray irradiation. In blood vessels, FPO@HMSNs-PAA accumulates around tumor tissues and enters into tumor cells via endocytosis. FPO released from FPO@HMSNs-PAA to produce H_2_O_2_ and O_2_ in the tumor acidic microenvironment. Under soft-X-ray irradiation, reactive oxygen species (ROS) level enhances with the generation of ROS, which can cause damage to DNA and further lead to tumor cells death.

In conclusion, formamide peroxide (FPO) with nontoxicity and high reactive oxygen content could enhance the oxygen concentration in tumor cells, which will improve the radio-sensitivity of soft-X-ray. The mechanism that FPO induced tumor cells death with soft-X-ray has been shown in Scheme 2. FPO was synthesized in the cavity and the channel of polyacrylic acid (PAA)-coated hollow mesoporous silica nanoparticles (FPO@HMSNs-PAA), which provides many advantages, involving enhanced FPO stability, controlled FPO delivery, and more effective FPO intracellular uptake. When entering cancer cells, FPO@HMSNs-PAA can release FPO and further decompose into H_2_O_2_ and O_2_. This process can enhance the dissolved oxygen concentration in tumor tissues and generate a large amount of ROS under soft-X-ray irradiation, which results in DNA damage and directly induces cellular necrosis. When compared with the previous research, FPO@HMSNs-PAA with an enhanced oxygen content has obvious cytotoxicity under lower-intensity soft-X-ray irradiation. The experimental results in vitro suggest that FPO@HMSNs-PAA, combined with soft-X-ray irradiation, holds promise for enhancing the radiotherapy efficiency as well as reducing the damage to normal tissue.

## Figures and Tables

**Table 1:** Structure parameter, FPO-loading efficiency and zeta potential analyses of the synthesized samples (HMSNs, HMSNs-NH_2_ and FPO@HMSNs-PAA).

Sample ^a^	S_BET_ (cm^3^ g^−1^)	V_pore_ (cm^3^ g^−1^)	D_pore_ (nm)	FPO Loading Content	Zeta Potential ^b^ (mV)
HMSNs	1166	0.84	2.7	/	−33.7 ± 1.3
HMSNs-NH_2_	1033	0.71	2.5	/	+36.1 ± 3.1
FPO@HMSNs	282	0.28	2	54.90%	−42.4 ± 1.9
FPO@HMSNs-PAA	94	0.13	/	38.32%	−56.5 ± 2.5

^a^ Data are presented as means ± SDs (*n* = 3). ^b^ Zeta potential were measured in PBS (pH = 7.40) at room temperature.

## Supplementary Materials

The following are available online at http://www.mdpi.com/1996-1944/11/4/596/s1, Figure S1: The cytotoxicity of formamide.
