# Supplementary material for: Efficient Active Oxygen Free Radical Generated in Tumor Cell by Loading-(HCONH2)·H2O2 Delivery Nanosystem with Soft-X-ray Radiotherapy
Source: Materials (Basel). 2018 Apr 12;11(4):596. doi: 10.3390/ma11040596 (PMC5951480; doi:10.3390/ma11040596)
Supplement: Supplementary file 1 [file materials-11-00596-s001.pdf]

materials

Supplementary Materials

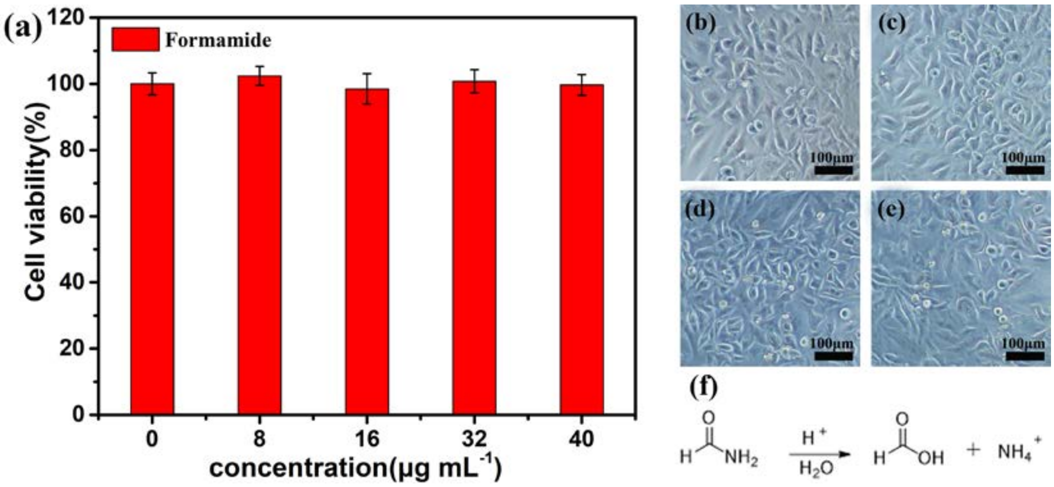

Figure S1 The cell viability of formamide (a); The Microscopy images of 4T1 cells incubated with formamide at the concentration of 0 (b), 8 (c), 16 (d) and 32 μg mL^{-1} (e); The chemical reactions of formamide in acidic conditions (f).

© 2018 by the authors. Submitted for possible open access publication under the terms and conditions of the Creative Commons Attribution (CC BY) license (http://creativecommons.org/licenses/by/4.0/).
